# Self- and Other-Directed Ageism and Worries Concerning COVID-19 Health Consequences

**DOI:** 10.1093/geroni/igab046.2268

**Published:** 2021-12-17

**Authors:** Liat Ayalon, Ella Cohn-Schwartz

**Affiliations:** 1 Bar Ilan University, Ramat Gan, HaMerkaz, Israel; 2 Ben-Gurion University, Beer-Sheva, HaDarom, Israel

## Abstract

Worries associated with COVID-19 health consequences are well-justified. They may motivate people to take safety precautions, but may hinder if they become too intense. Current research examined mainly age and gender as potentially associated with worries. This study instead, focuses on self-perceptions of ageing (SPA) and perceived age discrimination as potential predictors of worry, in light of the ageism pandemic which co-occurred with the COVID-19 outbreak. The study is based on a national representative sample of 1,092 adults aged 50+ in Israel. Phone interviews were conducted between March – May 2020, when Israel gradually moved from strict to partial lockdown. Our findings show that SPA and age-based discrimination in the healthcare system were significant predictors of worries. The findings point to the potentially negative impact of the ageism pandemic in relation to worries. Interventions that address ageism directed by self or others might alleviate people's worries in the COVID-19 pandemic.

